# Retraction Note to: Exosomal SNHG16 secreted by CSCs promotes glioma development via TLR7

**DOI:** 10.1186/s13287-022-02842-y

**Published:** 2022-04-13

**Authors:** Ruijie Zhang, Peng Li, Heli Lv, Nana Li, Suliang Ren, Wentao Xu

**Affiliations:** 1 Department of Neurology, Heze Hospital of Traditional Chinese Medicine, Heze, 274000 Shandong China; 2 Department of Neurosurgery, Heze Hospital of Traditional Chinese Medicine, 1036, Danyang Road, HezeShandong, 274000 China; 3 Department of Non-Treatment, Wenshang County Hospital of Traditional Chinese Medicine, Jining, 272501 Shandong China

## Retraction Note: Stem Cell Research & Therapy (2021) 12:349 10.1186/s13287-021-02393-8

The Editors-in-Chief have retracted this article at the request of the Corresponding Author.

After publication, the authors identified flaws in the study design and the data.

Further assessment of the article identified the following issues:

- The number of tumor cells inoculated into mice for the xenograft experiment was higher than recommended by internationally accepted standards.
- In Fig. 2d, the western blot image backgrounds and empty spaces in lane 1 appear remarkably similar.
- In Fig. 6c and d, a number of tumor images appear remarkably similar.

In addition, the authors were unable to locate the original ethics approval documents.

The Editors-in-Chief therefore no longer have confidence in the presented data and the conclusions of this article.

All authors agree to this retraction.

